# The in vitro and ex vivo effect of Auranta 3001 in preventing *Cryptosporidium hominis* and *Cryptosporidium parvum* infection

**DOI:** 10.1186/s13099-017-0192-y

**Published:** 2017-08-31

**Authors:** Alexandros Ch Stratakos, Filip Sima, Patrick Ward, Mark Linton, Carmel Kelly, Laurette Pinkerton, Lavinia Stef, Ioan Pet, Tiberiu Iancu, Gratiela Pircalabioru, Nicolae Corcionivoschi

**Affiliations:** 1 Bacteriology Branch, Veterinary Sciences Division, Agri-Food and Biosciences Institute, Newforge Lane, Belfast, BT9 5PX Northern Ireland, UK; 2 School of Biology, University of Bucharest, Splaiul Independentei 91-95, Bucharest, Romania; 3 Auranta, NovaUCD, Belfield Innovation Park, Belfield, Dublin 4, Ireland; 4 Banat’s University of Agricultural Sciences and Veterinary Medicine, King Michael I of Romania, Calea Aradului 119, 300645 Timisoara, Romania; 5 Research Institute of University of Bucharest, 36-46 Bd. M. Kogalniceanu, 5th District, 050107 Bucharest, Romania

**Keywords:** *Cryptosporidium hominis*, *Cryptosporidium parvum*, Infection, Natural antimicrobials

## Abstract

**Background:**

*Cryptosporidium* is a major cause of diarrhea worldwide in both humans and farm animals with no completely effective treatment available at present. In this study, we assessed the inhibitory effect of different concentrations of Auranta 3001 (0.1, 0.5 and 1%), a novel natural feed supplement, on *C. hominis* and *C. parvum* invasion of human ileocecal adenocarcinoma (HCT-8), bovine primary cells and *C. parvum* invasion of HCT-8, bovine primary cells and bovine intestinal biopsies. The effect of the feed supplement on the production of pro-inflammatory cytokines IL-8 and INF-γ, the anti-inflammatory cytokine IL-10, the expression of CpSUB1 protease gene during infection was also assessed by quantitative PCR (q-PCR). Transepithelial electrical resistance (TEER) was employed to measure the integrity of tight junction dynamics of the culture models.

**Results:**

Pre-treatment of intestinal cells or oocysts with the Auranta 3001 significantly reduced the invasiveness of *C. hominis* and *C. parvum* against HCT-8 and bovine primary cells in a dose dependent manner. The most pronounced reduction in the invasiveness of both parasites was observed when Auranta 3001 was present during infection. Levels of IL-8 were significantly reduced in both HCT-8 and bovine primary cells, while the levels of INF-γ and IL-10 showed opposite trends in the two cell lines during infection in the presence of Auranta 3001. CpSUB1 gene protease expression, which mediates infection, was significantly reduced suggesting that this enzyme is a possible target of Auranta 3001.

**Conclusions:**

Although, *C. hominis* and *C. parvum* use different invasion mechanisms to infect cells, the novel feed additive can significantly attenuate the entry of *Cryptosporidium* in HCT-8 cells, primary bovine cells and bovine intestinal biopsies and thus provide an alternative method to control cryptosporidiosis.

## Background

*Cryptosporidium* is a protozoan parasite of public health and veterinary significance that causes gastroenteritis in a range of vertebrate hosts. The majority of human infections are attributed to *C. hominis*, which humans as the only natural hosts and *C. parvum* which infects mostly mammals, including humans [1]. The infection is transmitted mainly by the fecal–oral route, and is initiated when sporozoites are released from oocysts present in water or food or by direct contact with an infected person or animal [2]. *Cryptosporidium* infection ultimately results is epithelial cell death thus leading to villous atrophy, malabsorption and distorted intestinal permeability, all of which contributes to the occurrence of watery diarrhea [3]. The host’s immune state has a key role in determining susceptibility to infection as well as the severity of cryptosporidiosis. In healthy people, cryptosporidiosis is self-limiting and gastrointestinal symptoms usually resolve spontaneously within 1–2 weeks, although asymptomatic carriage might occur. Nevertheless, in immunocompromised patients, severe watery diarrhea can develop which can lead even to death [4]. In tropical countries, *Cryptosporidium* caused diarrhea can result in impaired childhood development [5]. Numerous human cryptosporidiosis outbreaks have been identified on a global scale. The most extensive outbreak was recorded in 1993 in Milwaukee, U.S.A., during which approximately 400,000 people were infected with *Cryptosporidium* oocysts by drinking contaminated water. An increase in *Cryptosporidium* infections was also reported in Germany, United Kingdom and the Netherlands in 2012 [6]. In Asia and Africa, cryptosporidiosis is now considered the second most common cause of diarrhea [7]. Even though promising chemotherapeutic treatments against cryptosporidiosis are beginning to appear, no consistently effective treatments exist for humans and animals [8, 9]. Paromomycin and Nitazoxanide have been used but results showed limited efficacy against cryptosporidiosis and sometimes relapse of diarrhea has been observe [10, 11]. Halofuginone lactate is another anticoccidial agent used against cryptosporidiosis and although it diminishes oocyst shedding and the severity of the diseases, in calves, it does not result in a complete cure [12, 13].

Considering the potential side effects of and resistance to many drugs, attention has shifted towards plant extracts. Plants represent an excellent source of bioactive compounds and have a long history in the prevention as well as treatment of a range of human and animal diseases [14]. Therefore, interest in natural products with antiparasitic properties has increased in recent years. Curcumin has showed promising effects against *C. parvum* in vitro [15]. Also, the ethanolic extract from olive pomace has been shown to effectively inhibit *C. parvum* development [14]. Therefore, there is a great need to develop new anti-cryptosporidial agents, in order to provide an alternative method to control cryptosporidiosis. The present study aimed at investigating the antiparasitic effectiveness of the experimental natural feed supplement Auranta 3001 in the prevention *C. homins and C. parvum* infections in human and primary bovine cell models.

## Methods

### Parasites

*Cryptosporidium parvum* (G1) and *C. hominis* (G2) oocysts were obtained from American Type Culture Collection and stored in phosphate-buffered saline (PBS) at 4 °C until use. For cell culture assays oocysts were incubated for 15 min in sodium hypochlorite (4%) at 4 °C and washed three times with cold D-PBS (Dulbecco’s phosphate buffered saline, Sigma, UK) followed by centrifugation for 10 min at 2500×*g*. The pellet was suspended in 1 ml D-PBS and oocysts were counted in a haemocytometer. Prior to use, oocysts were un-treated or treated with 0.1, 0.5 or 1% Auranta A3001 and then washed five times with 20 mM PBS by centrifugation at 6000*g* for 3 min at 4 °C.

### HCT-8 epithelial cells and isolation of bovine gastrointestinal cells

Human ileocecal adenocarcinoma cells (HCT-8) were obtained from the American Type Culture Collection and maintained as previously described [16]. Bovine cells were prepared as previously described [17]. Briefly small duodenal tissue was placed in Dulbecco’s modified Eagle’s medium **(**DMEM, Sigma, UK) media containing 10% FBS (Fetal Bovine Serum), 100 μg of streptomycin/ml, 100 U of penicillin/ml, and 2.5 μg of amphotericin B (Sigma, UK)/ml. The tissue was incubated at 37 °C for 10 min with vigorous shaking. The supernatant was removed, and the tissue was placed in DMEM media containing 0.05% (wt/vol) collagenase (Sigma) and incubated at 37 °C for 15 min with vigorous shaking. The isolated cells and crypts were kept on ice until 500 μl was plated in 24-well Costar culture plates on 13-mm plastic coverslips with Dulbecco modified Eagle medium-Ham F-12 plus 10% (vol/vol) FBS, 8 μg of insulin/ml, 10 μg of gentamicin/ml, 50 μg of hydrocortisone/ml, 100 μg of streptomycin/ml, 100 U of penicillin/ml, and 2.5 μg of amphotericin B/ml.

### Infection of HCT-8 cells and primary—bovine intestinal cells

A concentration of 1.5 × 10^7^ *Cryptosporidium* oocysts (G1 or G2) was added to the isolated bovine cells or cultured HCT-8 cells as previously described [18]. There were three types of infection assays performed in order to test the efficiency of this antimicrobial product. First, the oocysts were pre-treated with Auranta A3001 (0.1, 0.5 and 1%), for 1 h, and washed in maintenance media prior to cell infection, secondly the cells were treated for 1 h prior the infection assays or the antimicrobial was present in the media during infection. For infection, the medium was removed from the host cell culture and replaced with 5 ml of oocyst suspension. As positive control, 1 μg/ml cytochalasin D was used as pre-treatment of cells before infection. The flasks were placed at 37 °C in a 5% CO_2_–95%. After incubation for 3 h, the infected cells were washed with D-PBS followed by the addition of 2 ml growth medium containing 100 U of penicillin/ml and 100 g of streptomycin/ml was added. Primary cells and HCT-8 cells were infected for up to 18 h. During this time period the cells remained viable and did not appear to be damaged by infection. The parasites were detected and counted in monolayers using lectin VVL staining as previously described [17]. After incubation for 4 h at 37 °C in 5% CO_2_ to allow attachment and penetration of sporozoites, the monolayers were washed with DMEM to remove non-invasive sporozoites, residual oocysts and non-adherent epithelial cells, and 5 ml of new growth medium with or without antimicrobial agents was added. Infected cell cultures were kept at 37 °C in 5% CO_2_. Experiments were performed in triplicates and expressed as the mean percentage of cells infected. The bovine biopsies were obtained from a local abattoir and infected following same procedure. Infected biopsies were fixed in formalin for 24 h and snapped freeze in liquid nitrogen. The IL-8, INF-γ and IL-10 supernatant concentration for each well was determined, at 18 h, by using a commercially available enzyme-linked immunosorbent assay systems from Quantikine, R&D Systems at 1% Auranta 3001. Results of cytokine concentration were expressed as pg (pictograms) per milliliter of culture medium. Each study run included wells with no parasite in the HCT-8 cell or bovine culture assay. All samples were analyzed in triplicate.

### q-PCR

*Cryptosporidium* G1 and G2 oocysts (2 × 10^5^ per flask) were used to infect confluent HCT-8 cell monolayers or isolated bovine intestinal cells grown in T-25 tissue culture flasks. Total RNA was isolated from infected and uninfected HCT-8 cells at 0, 6, 12 and 18 h postinfection, using an RNeasy kit (Qiagen-UK) and was reverse transcribed to cDNA by using the Superscript first-strand system (Roche, UK). cDNA was amplified by PCR for 35 cycles (94 °C for 1 min, 64 °C for 1 min, and 72 °C for 1.5 min), using primers (CpSUB1-F-AAAGGATCTGGAGTATATTAT and CpSUB1-R-AATATTTAATGTCCCAGAGG) [16]. Uninfected cells were used as control. The PCR reactions were set using SYBR Green Master mix (Applied Biosystems, UK).

### TEER

To investigate the effect of *C. parvum* G1 and G2 on the barrier properties HCT-8 cells and isolated bovine cells, transepithelial electrical resistance (TEER) was measured at 3 h postinfection using an EVOM X meter (World Precision Instruments) coupled to an Endohm chamber (World Precision Instruments), and the infected cells compared to noninfected cells whose TEER was also measured at 3 h following infection.

## Results

### In vitro and ex vivo effect of Auranta 3001 on invasiveness of *C. hominis* and *C. parvum* to HCT-8 cells, primary bovine intestinal cells and bovine intestinal biopsies

**Fig. 1:**
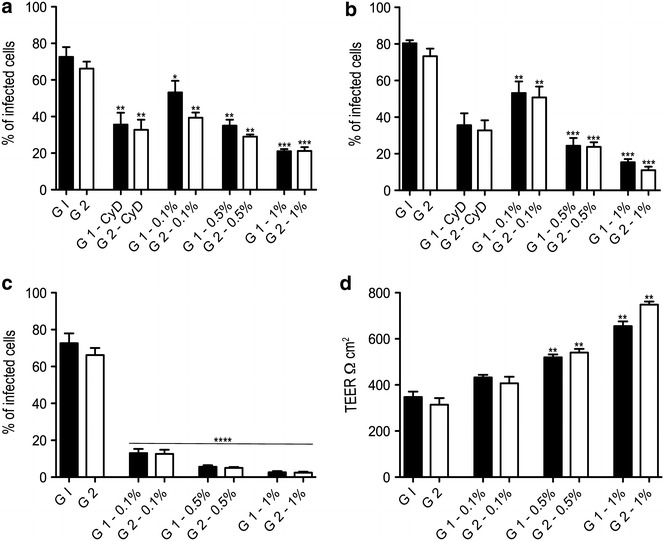
Effect of different concentrations of Auranta 3001 on *Cryptosporidium* invasiveness in HCT-8 cells. **a** HCT-8 cells exposed to different concentrations of Auranta 3001 for 1 h prior to infection. **b** *Cryptosporidium* oocysts exposed to different concentrations of Auranta 3001 for 1 h prior to infection. **c** Auranta 3001 present in the medium during infection. **d** Transepithelial electrical resistance (TEER) values of non-treated cells and cells treated with Auranta 3001. *Asterisks* indicate significant differences (*p < 0.05; **p < 0.01; ***p < 0.001). *Error bars* represent the standard deviation of means from three different experiments. G1, *Cryptosporidium hominis*; G2, *Cryptosporidium parvum*; CyD, cytochalasin D

**Fig. 2:**
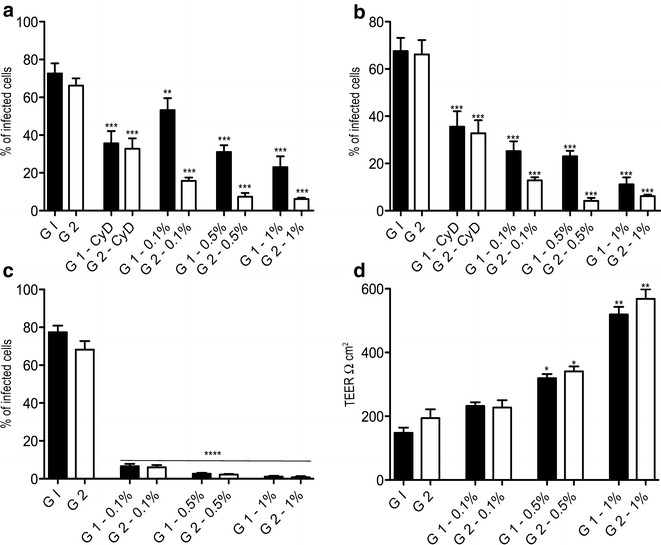
Effect of different concentrations of Auranta 3001 on *Cryptosporidium* invasiveness in primary bovine cells. **a** Primary bovine cells exposed to different concentrations of Auranta 3001 for 1 h prior to infection. **b** *Cryptosporidium* oocysts exposed to different concentrations of Auranta 3001 for 1 h prior to infection. **c** Auranta 3001 present in the medium during infection. **d** Transepithelial electrical resistance (TEER) values of non-treated cells and cells treated with Auranta 3001. *Asterisks* indicate significant differences (*p < 0.05; **p < 0.01; ***p < 0.001). *Error bars* represent the standard deviation of means from three different experiments. G1, *Cryptosporidium hominis*; G2, *Cryptosporidium parvum*; CyD, cytochalasin D

**Fig. 3:**
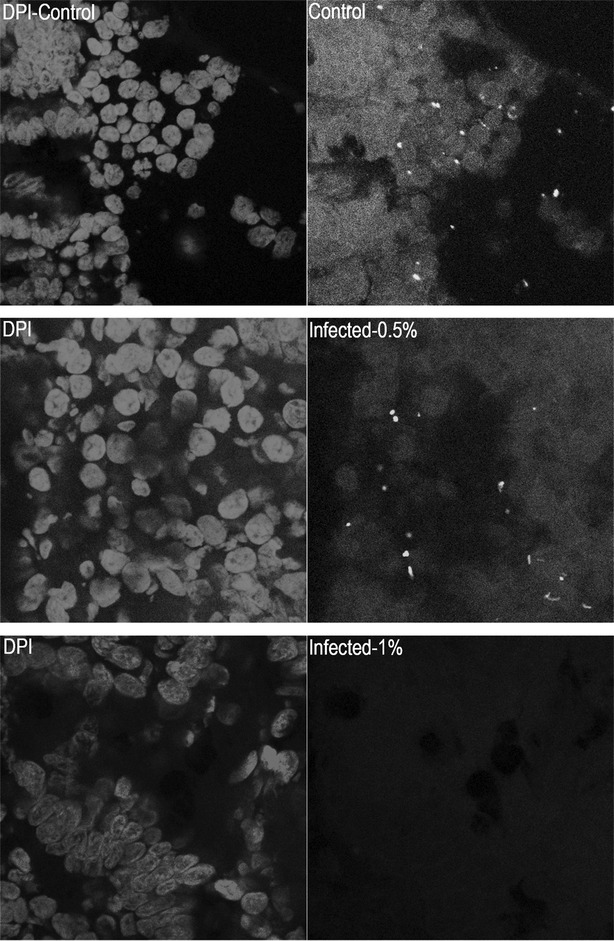
Lectin VVL staining of bovine intestinal biopsies infected with *C. parvum*. Effect of different concentrations of Auranta 3001 on *C. parvum* ex vivo infection of bovine intestinal biopsies

The invasiveness of *C. hominis* (G1) and *C. parvum* (G2) was quantified. Pre-treatment of host cells with cytochalasin D (CyD) significantly reduced invasion of *C. hominis and C. parvum* into human and primary bovine cells (Figs. 1, 2) due to disruption of the host actin cytoskeleton. Results from the invasion assays showed that both parasites were able to invade HCT-8 cells and primary bovine intestinal cells with a similar efficiency (Figs. 1, 2). Pre-treatment of HCT-8 cells with different concentrations of the novel feed supplement reduced invasion of primary bovine cells and HCT-8 cells by *C. hominis* and *C. parvum* in a dose dependent manner. The same potent inhibition of infection was observed when of oocysts of both parasite species were pre-treated with increasing concentrations of Auranta 3001. Comparing the invasiveness of the two parasites in the bovine cell model, it is noteworthy that in both the pre-treatment of intestinal cells and oocysts, *C. parvum* invasiveness was significantly lower compared to the invasiveness of *C. hominis.* This phenomenon was not observed with the HCT-8 cells with both parasites showing similar invasiveness. To extend the above observations, invasiveness efficiency of both parasites was determined, without pre-treatment, when the feed supplement was present during infection. In this case, the effectiveness of Auranta 3001 in decreasing *Cryptosporidium* invasiveness was even more pronounced, reaching approximately 60–70%, even at the lower supplement concentrations (0.1%). Similar effects of Auranta 3001 were observed when the *C. parvum* infection of bovine intestinal biopsies was investigated (Fig. 3) suggesting clearly its potential in reducing the virulence of this parasite.

### Protection against disassembly of cellular tight junctions by Auranta 3001

The transepithelial electrical resistance (TEER) was measured to assess the changes in the integrity of tight junctions and permeability in the cell monolayers. Figures 1d and 2d show that low TEER values were obtained after a 3 h infection, which suggests dysfunction of the epithelial barrier during the in vitro intestinal infections with both parasites and both epithelial cell models. However, when Auranta 3001 was present in the medium during infection a significant increase in TEER was observed. Importantly, TEER values increased as the concentration of the feed supplement increased.

### Effect of Auranta 3001 on the levels of IL-8, IFN-γ and IL-10

**Fig. 4:**
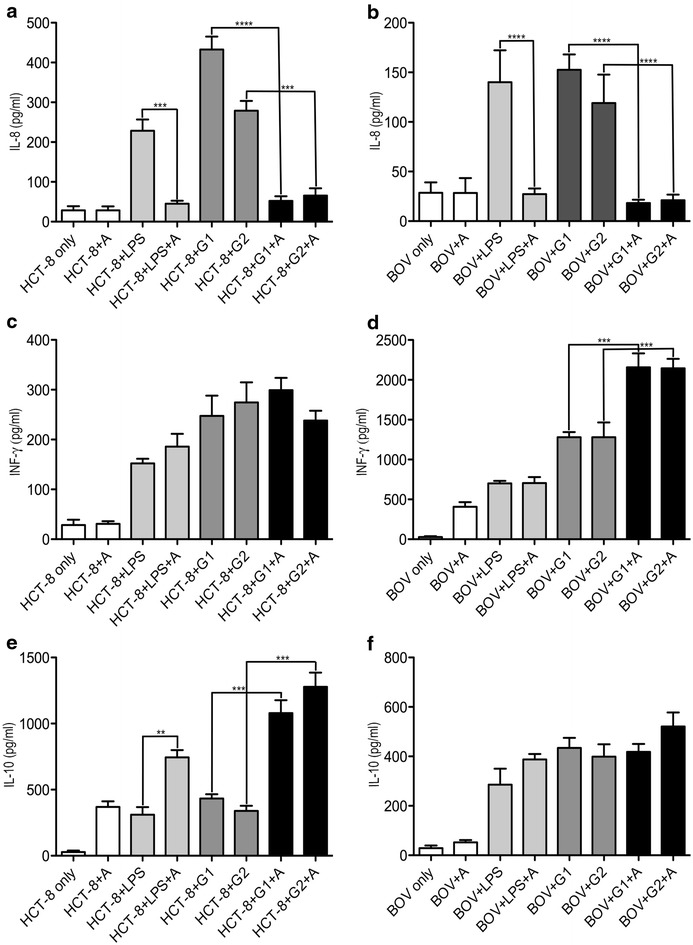
Cytokine expression in HCT-8 and primary bovine intestinal cells during infection with LPS and *C. hominis* and *C. parvum*. All of the cells were cultured to 80–90% confluency and challenged (infected) with *C. hominis* and *C. parvum* for 18 h. **a** IL-8 levels of HCT-8 cells. **b** IL-8 levels of primary bovine cells. **c** IFN-γ levels of HCT-8 cells. **d** IFN-γ levels of primary bovine cells. **e** IL-10 levels of HCT-8 cells (**f**) IL-10 levels of primary bovine cells. LPS treated HCT-8 and primary bovine cells were used as positive controls. Not treated HCT-8 and primary bovine were used a negative controls. *Asterisks* indicate significant differences (***p < 0.001; ****p < 0.0001). *Error bars* represent the standard deviation of means from three different experiments. G1, *Cryptosporidium hominis*; G2, *Cryptosporidium parvum*

In this study, three different types of cytokines (IL-8, IFN-γ and IL-10) where investigated. Bacterial cell wall lipopolysaccharide (LPS) was used as a positive control because this endotoxin is a prototypic microbial trigger that stimulates innate immunity. The resultant inflammatory responses are essential in early host defense but may also contribute to later organ injury [19]. The cytokine IL-8 levels were examined after an 18 h incubation (Fig. 4a, b). In HCT-8 cells, the secretion, in vitro, showed decreased IL-8 levels (approx. 350 pg/ml reduction for *C. hominis* (*p* < 0.0001) and approx. 250 pg/ml for *C. parvum* (p ≤ 0.001)) when the cells were treated with Auranta 3001. In primary bovine intestinal cells, the same trend was observed, with the IL-8 levels being reduced by more than 100 pg/ml for both parasites (*p* < 0.0001). Similar results were obtained with LPS treated HCT-8 and primary bovine cells. The levels of the immunomodulatory cytokine IFN-γ (Fig. 4c, d) were not influenced by the feed supplement in HCT-8 cells, whereas in primary bovine cells, the levels of IFN-γ increased by approximately 900 pg/ml (*p* < 0.001) for both HCT-8 and primary bovine infected cells. LPS treatment increased levels of IFN-γ in both cell models but Auranta 3001 did not have an effect on the levels of IFN-γ. The immunosuppressive cytokine IL-10, (Fig. 4e, f) showed a pronounced increase in HCT-8 cells, when they were incubated with Auranta 3001, (*p* < 0.0001) showing that the feed supplement has a great capacity in suppressing the inflammatory process. In primary bovine cells the levels of IL-10 did not differ significantly between the control and the Auranta 3001 treated ones. LPS treated HCT-8 and primary bovine cells showed the same trend.

### Effect of Auranta 3001 on expression of CpSUB1, a subtilisin-like protease, in *C. parvum* during infection

**Fig. 5:**
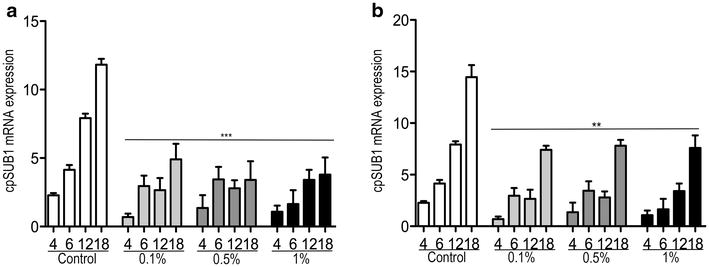
Effect of different concentrations of Auranta 3001 on CpSUB1 expression in *C. parvum* infected HCT-8 and primary bovine cells. **a** C*. parvum* infected HCT-8 cells (**b**) bovine primary cells. qPCR was performed on total RNA extracted from *C. parvum* cells at different time intervals during infection. *Asterisks* indicate significant differences (**p < 0.01; ***p < 0.001). *Error bars* represent the standard deviation of means from three different experiments

Figure 5a, b present the quantification of CpSUB1 expression at different times in *C. parvum* infected HCT-8 cells and primary bovine cells, respectively. qPCR of RNA from both cell models infected with *C. parvum* for increasing periods of time revealed that the CpSUB1 gene is expressed throughout infection. When HCT-8 cells and primary bovine cells were treated with increasing concentrations of the natural feed supplement, CpSUB1 gene expression was significantly reduced during the course of infection in vitro.

## Discussion

In order to tackle the issue of the lack of effective therapeutic agents against Cryptosporidiosis, we explored the efficiency of Auranta 3001, a natural feed supplement, against *C. hominis* and *C. parvum* in vitro. Preliminary results revealed that the feed supplement was toxic to HCT-8 bovine cells at concentrations higher than 1%. Lower concentrations had insignificant effects on both of the cell models and thus concentrations up to 1% were investigated.

It has been reported that different *Cryptosporidium* species utilise different mechanisms to infect cells of different origins [17] therefore anti-*Cryptosporidium* agents able to effectively prevent invasion in different host cells would be of great benefit. In the present study, *C. hominis* and *C. parvum* is able to effectively invade both HCT-8 and primary bovine cells. To evaluate the effect on parasite invasiveness, the feed supplement was applied at different concentrations to *C. hominis* and *C. parvum* oocysts for 1 h before infection. Pre-treatment of the oocysts resulted in statistically significant reductions (*p* < 0.01–0.001) in the invasiveness for both HCT-8 and bovine cells with the reduction being more pronounced with increasing supplement concentrations. It is noteworthy that although the invasiveness of *C. hominis* and *C. parvum* was similar for the HCT-8 cells, it showed differences in the bovine cells with *C. parvum* invasiveness being significantly lower compared to the HCT-8 cell line (Figs. 1a, 2a).

Potentially the novel feed supplement can also interfere with host cell metabolic pathways, which subsequently affect parasite infection and growth. To address this possibility, HCT-8 cells and bovine cells were pre-treated with the feed supplement for 1 h and then infected with *C. hominis* or *C. parvum* in the absence of the supplement. The pre-treatment of host cells with increasing concentrations of Auranta 3001 (0.1–1%), lead to a significant inhibition of invasiveness for both parasites (Figs. 1b, 2b). In this case too, *C. parvum* invasiveness in bovine cells was significantly lower compared to the HCT-8 cells.

Natural extracts have been employed in other studies against Cryptosporidium. Garlic juice (*Allium sativum*) has been shown to significantly reduce *Cryptosporidium* oocysts from stool of immunosuppressed mice without affecting intestinal architecture [10]. Curcumin, a natural polyphenolic compound, although does not reduce viability of *C. parvum* it can attenuate invasiveness in HCT-8 cells by 65% at a concentration of 200 μM [15]. Pomegranate extract (3.75%) supplemented in milk has also shown to reduce fecal oocyst count and diarrhea intensity and duration in neonatal calves [20]. Although *C. hominis* and *C. parvum* possess different pathways to infect different host cells [17], the results presented here revealed that the natural feed supplement is a potent inhibitor of *C. hominis* and *C. parvum* invasiveness in both human and primary bovine cells.

The cytokine profile of HCT-8 and primary bovine cells was also determined in order to investigate the host immune response to the natural feed supplement. The immune defence mechanism of an immunocompetent host against *Cryptosporidium* spp. used to recover from *Cryptosporidium* spp. infection involves both functional cellular and humoral immunities, but the actual mechanism is still unknown. Studies have shown that IL-8 is expressed after *Cryptosporidium* spp. in vitro infection of intestinal cell lines and human intestinal xenographs [21]. In the present study, exposure to Auranta 3001 significantly decreased the IL-8 levels, which suggests that Auranta 3001 is not recognized as a pathogenic signal by both HCT-8 cells and primary bovine cells indicating the anti-inflammatory role for Auranta 3001 in the gastro-intestinal tract inflammation during *Cryptosporidium* spp. infection. Also, IFN-γ results showed that Auranta 3001 did not have any effect on IFN-γ production in HCT-8 cells, keeping concentrations at similar levels with the controls, whereas in bovine primary cells, the levels of IFN-γ were significantly increased, showing its effectiveness in mediating the immune response in this type of cells when infected with *C. hominis* and *C. parvum*. Studies have shown that the immune response towards *Cryptosporidium* in humans differs from that in animals. IFN-γ production in mice seems to be linked with the innate and primary immune responses [22], whereas in humans it is probably related with the memory response towards the parasite. Even though IFN-γ has been shown to play an important role in both the innate and adaptive immune responses to *C. hominis* and *C. parvum*, the mechanisms of resistance mediated by this cytokine alone are not completely understood yet [21]. Interleukin-10 plays a major role in the resolution of inflammation during sepsis and infection but is also involved in persistence of pathogens by interfering with innate and adaptive immunity [23]. The Auranta 3001 results on HCT-8 cells, revealed its anti-inflammatory properties. The levels of IL-10 in HCT-8 cells increased significantly during infection with *C. hominis* and *C. parvum*. On the other hand, the supplement did not have any effect on IL-10 production in primary bovine cells, with the levels of this cytokine remaining at similar concentrations with the controls.

Parasite proteases are of particular importance since they are involved in the proteolysis of surface and apical proteins that mediate invasion [24, 25]. A number of these proteins are processed by serine proteases. Evidence from the use of protease inhibitors has suggested that serine proteases might be essential for host cell infection by *C. parvum* infection in vitro [26, 27].

Genes expressing serine proteases have been identified for *Cryptosporidium* [28, 29]. In order to better understand the antiparasitic mechanism of the natural feed supplement, in this study, we focused on the subtilisin-like serine protease CpSUB1 involved in the proteolytic cleavage of *Cryptosporidium* gp40/15 to produce the surface glycopeptides gp40 and gp15 which are involved in infection [27]. In the present study, qPCR results showed that the CpSUB1 gene in *C. parvum* is expressed throughout infection which is consistent with the study of Wanyiri et al. [16] in which a semi-quantitative determination was employed. Specific quantification of CpSUB1 gene revealed that when infection of both HCT-8 and primary bovine cells took place in the presence of different concentrations of the natural supplement expression was significantly reduced at all the concentrations used and at all time points investigated during infection (Fig. 5a, b). These results in combination with the fact that the natural supplement significantly inhibited *C. parvum* infection of HCT-8 cells in a dose-dependent manner compared to untreated controls shows that subtilisin-like serine protease CpSUB1 is targeted by the natural feed supplement leading to decreased invasion. However, it is possible that the natural supplement might exert an effect on other targets as well. Although, the expression of subtilase protease gene for *C. hominis* (ChSUB1) was not studied here, it has been shown that ChSUB1 and CpSUB1 are 98% identical with the alignment of the CpSUB1 and ChSUB1 nucleotide sequences identifying primarily silent substitutions [28]. Therefore, based on the significantly reduced invasiveness of *C. hominis* observed, we hypothesised that ChSUB1 is a target for the natural feed supplement as well.

Disruption of cell tight junctions is known to contribute to intestinal diseases by enteric pathogens [30]. TEER values are considered strong indicators of cellular barrier integrity [31]. In vitro studies have shown a rapid decrease in TEER as one of the characteristics of Cryptosporidiosis [32, 33]. In this study, infection with *C. hominis* and *C. parvum* resulted in low TEER values for both HCT-8 and bovine cells which corresponds to disrupted tight junction architecture and barrier function. However, in the presence of the novel supplement significantly higher TEER values were observed showing the protective effect of Auranta 3001 on both cell monolayers (Figs. 1d, 2d). Protein kinace C (PKC) mediates calcium-induced tight junction assembly and its inhibition has been found to stop the normal distribution of tight junction-associated proteins such as ZO-1 and cingulin [34]. A possible explanation of the reduced invasiveness observed is that exposure of HCT-8 and primary bovine cells to the feed supplement during infection caused stimulation of tight junctions in the host cells by inhibiting PKC although further studies are needed to confirm this.

## Conclusions

In conclusion, we found that the Auranta 3001 is able to significantly reduce the invasiveness of *C. hominis* and *C. parvum* and protect host cells in vitro. It was also shown that the natural feed supplement induced different host cell response in HCT-8 and primary bovine cells, and its activity is exerted, at least partly, by inhibiting CpSUB1 gene expression. Based on these results, Auranta 3001 shows great potential as a method to control cryptosporidiosis.
